# Fourth Ventricular Schwannoma: Identical Clinicopathologic Features as Schwann Cell-Derived Schwannoma with Unique Etiopathologic Origins

**DOI:** 10.1155/2011/165954

**Published:** 2011-12-13

**Authors:** Tiffany R. Hodges, Isaac O. Karikari, Shahid M. Nimjee, June Tibaleka, Thomas J. Cummings, Senthil Radhakrishnan, Allan H. Friedman

**Affiliations:** ^1^Division of Neurosurgery, Department of Surgery, Duke University Medical Center, P.O. Box 3807, Durham, NC 27710, USA; ^2^Department of Pathology, Duke University Medical Center, Durham, NC 27710, USA

## Abstract

*Background*. To our knowledge, this is the sixth reported case in the literature of fourth ventricular schwannoma. The etiology and natural history of intraventricular schwannomas is not well understood. A thorough review of potential etiopathogenic mechanisms is provided in this case report. *Case Description*. A 69-year-old man presented with an incidentally found fourth ventricular tumor during an evaluation for generalized weakness, gait instability, and memory disturbance. Magnetic resonance imaging (MRI) revealed a heterogeneously enhancing lesion in the fourth ventricle. A suboccipital craniotomy was performed to resect the lesion. Histopathological examination confirmed the diagnosis of schwannoma (WHO grade I). *Conclusions*. Schwannomas should be considered in the differential diagnosis of intraventricular tumors. Although the embryologic origins may be different from nerve sheath-derived schwannomas, the histologic, clinical, and natural history appear identical and thus should be managed similarly.

## 1. Introduction

Intraventricular schwannomas are rare tumors with only 17 cases reported in the literature [1–16]. The etiology of intraventricular schwannomas is not known but they are thought to arise from a separate mechanism from the commonly encountered intracranial schwannomas. Proposed origins include neoplastic transformation of previously displaced neural crest cells during embryogenesis, differentiation of multipotent mesenchymal cells into Schwann cells, stem cells, and neoplastic transformation from autonomic cells found in intrinsic arteries and the choroid plexus [2, 5, 6, 10, 12, 15–19].

We present a rare case of fourth ventricular schwannoma and provide a review of the current literature.

## 2. Case Report

### 2.1. Patient Presentation

A 69-year-old man presented with progressive worsening generalized weakness, gait instability, and memory disturbances. His neurological examination was unremarkable.

**Figure 1:**
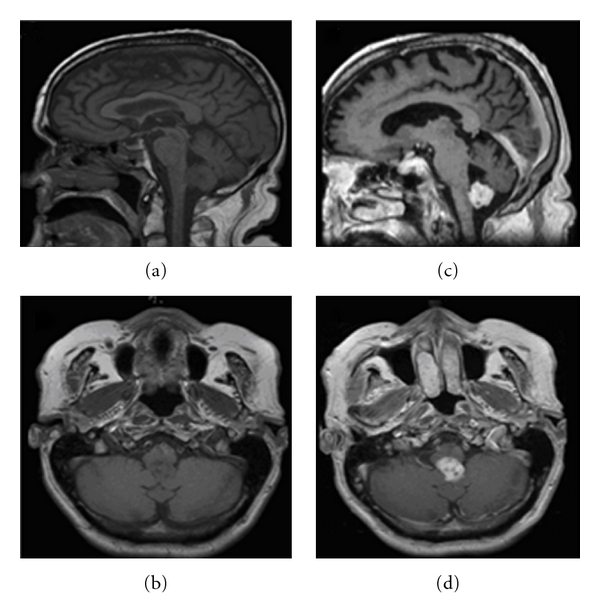
Sagittal and axial pre- and Postcontrast MR images. (a) Sagittal image without gadolinium showing a homogenous lesion in the fourth ventricle. (b) Axial image without gadolinium showing the lesion. (c) Sagittal postcontrast image showing a heterogeneous enhancement of the lesion. (d) Axial postcontrast also showing heterogeneous enhancement.

An MRI with contrast of the brain revealed a 1.8 cm × 1.8 cm × 2.0 cm heterogeneously enhancing lesion in the posterior fourth ventricle (Figure 1). Given the MRI characteristics and location of the tumor, the differential diagnoses included ependymoma, subependymoma, choroid plexus papilloma, and pilocytic astrocytoma.

### 2.2. Intraoperative Findings

A standard suboccipital craniotomy with telovelar approach was used to access the tumor. A firm and yellowish appearing tumor originating from the floor of the fourth ventricle was encountered. The inside of the tumor, however, was purple in color. The tumor did not appear to arise from any of the lower cranial nerves, and actually appeared to arise from the area postrema on the right side. A complete microsurgical resection was performed. 

### 2.3. Histological Findings

**Figure 2:**
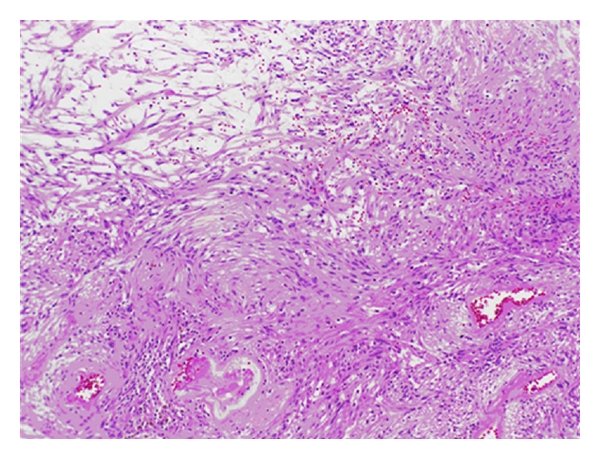
Schwannoma characterized by Antoni A and Antoni B architecture (hematoxylin and eosin ×10).

Microscopic examination showed a spindle cell neoplasm characterized by Antoni A and Antoni B areas with Verocay bodies characteristic of schwannomas (Figure 2). Areas of chronic inflammation, hemosiderin pigment deposition, and thickened blood vessels were seen. The tumor cells are immunopositive for S-100 protein and glial fibrillary acidic protein (GFAP). Epithelial membrane antigen (EMA) and neurofilament protein stains were negative. The MIB-1 index was low. Type 4 collagen was also weak.

## 3. Discussion

Intraventricular schwannomas are very rare tumors with only 17 previously reported cases in the literature [1–16] (see Table 1). The vast majority of reported cases have been located in the lateral ventricle with only 5 cases in the fourth ventricle [6, 12, 15, 16]. Intraventricular schwannomas have been reported in both pediatric and adult populations, and do not appear to have a predilection for a particular age. Most reported cases have been histologically benign and therefore surgical resection is considered curative except in two cases where a malignant schwannoma was found [8, 10].

The natural history and growth patterns of the intraventricular schwannoma, unlike its extra-axial counterpart, are not completely understood given the rarity of its occurrence. Based on the previously reported literature, this neoplasm appears to assume a benign course with no recurrence when there is no histological evidence of malignancy. Barbosa et al. described a case of cystic intraventricular schwannoma without recurrence for 10 years after resection [1]. Of the two reported cases of malignant intraventricular schwannomas, one was identified incidentally during autopsy [10] while the other patient had recurrence with drop metastases requiring reoperation and whole brain radiation [8].

Similar to other intracranial schwannomas, intraventricular schwannomas radiographically demonstrate heterogeneous or homogeneous contrast enhancement with cystic components and occasional calcification. It is often not considered in the differential diagnosis of fourth ventricular tumors due to the rarity of occurrence. It is usually difficult to identify intraventricular schwannomas on neuroimaging because it lacks specific imaging characteristics to differentiate them from other intraventricular tumors [1, 2, 9, 16]. In our case for example, the differential diagnoses consisted of ependymoma, subependymoma, choroid plexus papilloma, or pilocytic astrocytoma. Even for the experienced neurological surgeon, the intraoperative diagnosis can be difficult as the tumor is not associated with any cranial nerves.

As reported in 5 other cases of fourth ventricular schwannomas, a midline suboccipital craniotomy was implemented for surgical access to the lesion. Most of these tumors arise from the ventricular floor, but in 2 reported cases, the tumor appeared to originate from the roof of the fourth ventricle [6, 12]. In addition, surgical removal of these fourth ventricular tumors was curative with no need for further treatment. Since these tumors are located within the fourth ventricle, a possible accompanying feature of these tumors is hydrocephalus with subsequent need for a ventriculostomy, shunt, or endoscopic fenestration. This, however, is rare, and was not encountered in our patient.

The histological appearance of intraventricular schwannomas is similar to the commonly encountered extra-axial schwannomas, characteristically demonstrating spindle cell architecture with Antoni A and B areas. Antoni A areas are highly cellular with nuclear palisading and associated Verocay bodies. Antoni B areas consist of loosely organized tissue with myxomatous and cystic changes. The proliferation marker MIB-1 is typically low, although focal areas of high MIB-1 activity have been detected in some neuropathological specimens of intraventricular schwannomas [12]. Immunohistochemical analysis is also positive for S-100 and GFAP and negative for EMA and neurofilament protein [12]. 

Despite this similar histological appearance, intraventricular schwannomas and extra-axial schwannomas may differ in their source of origin. It is well accepted that extra-axial schwannomas are derived from the myelinated cell of the peripheral nervous system [20]. Schwannomas typically remain attached to the parent nerve while growing within a capsule, and usually involve the vestibular branch of cranial nerve VIII or the dorsal roots of the spinal cord [20]. In contrast, nerve fibers of the central nervous system are not encased by a Schwann cell sheath; therefore, the occurrence of a schwannoma within the central nervous system is surprising.

Interestingly, the exact source of origin for intraventricular schwannomas is currently not known, as there are several proposed etiopathogenic mechanisms. One such theory is neoplastic transformation of neural crest cells that were displaced during embryogenesis [2, 4, 5, 9, 15, 16]. Schwann cells are derived from the neural crest, and it is possible that a few of these cells could become trapped within the neural tube as it closes and undergoes differentiation during embryonic development. These neural crest cells may deposit in the ventricle, and neoplastic transformation of these Schwann cells could result in a schwannoma in an unusual location. This theory could explain why these tumors are so rare [15], and could also explain why our patient presented with an intraventricular schwannoma.

Another hypothesis is transformation of autonomic nervous tissue accompanying intrinsic arteries and choroid plexus [1, 2, 6, 9, 11, 13, 15, 16]. This is supported by the identification of nerve fibers in the choroid plexus of the fourth ventricle by Benedickt in 1874 and Stöhr in 1922 [21, 22]. Since autonomic nerves are invested with a Schwann cell covering, it is possible that intraventricular schwannomas may arise from neoplastic transformation of autonomic nervous tissue within the choroid plexus. Considering that the lesion in our patient appeared to arise from the area postrema intraoperatively and not from the choroid plexus, this theory is unlikely.

Another proposed theory is schwannomas arising from aberrant peripheral nerve fibers. This proliferation may arise from localized traumatic injury; for example, focal axonal proliferation in the pons adjacent to infarcted brain tissue [23] or traumatic schwannomas associated with spinal cord compression [24]. It should be noted that these may not be true neoplasms, but rather regenerative proliferation of injured peripheral nerve found within the central nervous system. Our patient did not have a traumatic injury associated with his intraventricular schwannoma, so this theory is unlikely in our case. 

Others have proposed transformation of multipotent cells into Schwann cells and proliferation of foci of Schwann cells (“schwannosis”) as potential etiologies [5, 7, 16]. This multipotent cell theory is based upon the idea that the Schwann cell, a specialized mesenchymal element, is derived from primitive multipotent cells that can give rise to other types of specialized mesenchymal elements in the appropriate cellular milieu [25]. Similarly, the term “schwannosis” refers to hamartomatous lesions consisting of Schwann cells and reticulin fibers that are formed from the conversion of pial mesodermal cells into Schwann cells [26]. Schwannomas can result from neoplastic proliferation of these pial mesodermal cells. This theory could also potentially explain the etiopathologic origin of the intraventricular schwannoma in our case.

Lastly, there is now much evidence that cancer stem cells may be responsible for maintenance and growth of tumors. The cancer stem cell hypothesis centers on the idea that tumors arise from a small subpopulation of stem cells, which have the ability to self-renew, produce daughter tumorigenic cells, and can give rise to nontumorigenic cancer cell phenotypes. Stem cells, for example, have been detected in neural tumors, including glioblastoma, medulloblastoma, and ependymoma [18, 19]. These cells have the ability to reform parent tumor when implanted into nude mice and can partially differentiate in vivo. It has been shown that there is upregulation of certain stem cell markers, including nestin and CD44, in some vestibular schwannomas [17]. In this study, 92% of human vestibular schwannomas demonstrated an upregulation of CD44 at the mRNA level compared to normal human Schwann cells [17]. Further studies, however, are necessary to determine whether neural stem cells play an important role in schwannoma tumorigenesis. 

## 4. Conclusions

Fourth ventricular schwannomas are rare. Although their etiopathologic origin is different from extra-axial schwannomas, their imaging, histology, ability to achieve a gross total resection, and clinical course appear identical with other schwannomas and should be managed similarly. Although rare, schwannomas should be considered in the differential diagnosis of contrast-enhancing fourth ventricular lesions in both children and adults.

## Figures and Tables

**Table 1:** Summary of reported cases of intraventricular schwannoma.

Reference	Age/sex	Location (ventricle)	Extent of resection	Recurrence/progression of residual tumor
Dow et al. [4]	43/M	Fourth	NS	NS
Ghatak et al. [7]	15/M	Right lateral	Total	No
Marchand et al. [10]	63/F	Right lateral	Total	No
Messing-Jünger et al. [11]	8/M	Right lateral	Total	No
Lévêque et al. [9]	44/M	Left lateral	Total	No
Benedict et al. [2]	7/M	Fourth	Subtotal	No
David et al. [3]	61/M	Fourth	Subtotal	No
David et al. [3]	78/F	Fourth	Subtotal	No
Estrada Mastache et al. [6]	40/M	Right lateral	Subtotal	Yes (death)
Feigin [25]	13/F	Right lateral	Total	No
Erdogan et al. [5]	36/F	Fourth	Total	No
Benedickt [21]	21/M	Right lateral	Total	No
Jung et al. [8]	16/M	Right lateral	Total	No
Koeppen et al. [24]	21/F	Third	Total	No
Galli et al. [18]	16/M	Right lateral	Total	No
Barbosa et al. [1]	15/M	Right lateral	Subtotal	No
Ahmad et al. [17]	71/F	Fourth	Total	No
Present case	69/M	Fourth	Total	No
